# Bioelectrical impedance compared to computed tomography for muscle mass evaluation in critically ill elderly patients

**DOI:** 10.62675/2965-2774.20260004

**Published:** 2026-07-02

**Authors:** Sandra Regina Alves Belo, Naiara Lima Matos, Elisa Colonnezi, Leandro Utino Taniguchi

**Affiliations:** 1 Instituto Sírio-Libanês de Ensino e Pesquisa, Hospital Sírio-Libanês Postgraduate Program in Health Sciences São Paulo SP Brazil Postgraduate Program in Health Sciences, Instituto Sírio-Libanês de Ensino e Pesquisa, Hospital Sírio-Libanês - São Paulo (SP), Brazil.

Computed tomography (CT) is considered a gold standard for muscle mass quantification in the recent sarcopenia consensus^(1)^ and has been associated with mortality in critically ill elderly patients.^(2)^ However, it is not feasible to apply to all patients admitted to the intensive care unit (ICU). Bioelectrical impedance analysis (BIA) is a bedside tool for body composition evaluation^(3)^ that has been correlated with CT muscle mass in ICU patients.^(4)^ However, older age has been demonstrated as a factor associated with BIA and CT discordance for muscle mass assessment.^(5)^ Therefore, we aimed to study the correlation between BIA and muscle area measured on CT and the accuracy of BIA in discriminating elderly ICU patients with adequate muscle area on CT.

## METHODS

This was a diagnostic cross-sectional study performed in the general medical-surgical intensive care units of *Hospital Sírio Libanês* (São Paulo, Brazil). We analyzed all patients aged ≥ 65 years who underwent a CT scan for clinical reasons during ICU admission, did not meet any exclusion criteria, and were admitted between December 2021 and June 2022 (Supplementary Material). The study was approved by the Ethics Committee of *Hospital Sírio-Libanês*, which waived informed consent. The present report followed the STARD 2015 statement.^(6)^

Computed tomography scans were analyzed by a trained investigator at the level of the 12th thoracic vertebra (T12), unaware of BIA results, using CoreSlicer^®^ (https://coreslicer.com).^(7)^ Bioelectrical impedance analysis evaluation was performed by trained professionals unaware of CT results with a portable BIA device for segmental BIA (InBody S10^®^, Biospace Co. Ltd, South Korea). The correlation between CT and BIA-derived parameters was estimated using the Spearman correlation method. The discrimination of BIA-derived parameters for identifying patients with adequate muscle mass area at T12 was evaluated using area under the Receiver Operating Characteristic curve (AUROC) analysis (Supplementary Material).

## RESULTS

A total of 48 patients (median age of 81 years, Table 1S - Supplementary Material) were included (Figure 1S - Supplementary Material). More than half of the patients had low skeletal muscle area at the T12 level by CT scan analysis (Table 2S - Supplementary Material). There was a positive and significant correlation between CT muscle area and BIA-derived variables. However, the majority of these parameters were not discriminative (Table 1), except for phase angle and MMTalluri.

**Table 1:** Discrimination of bioelectrical impedance analysis-derived variables for adequate skeletal muscle area at T12 computed tomography

Variable	Spearman correlation	AUROC (95%CI)
Lean body mass	0.72 (p < 0.001)	0.59 (0.43 - 0.76)
Skeletal muscle mass	0.74 (p < 0.001)	0.61 (0.45 - 0.77)
Muscle Mass Talluri	0.72 (p < 0.001)	0.67 (0.51 - 0.83)*
Phase angle	0.36 (p = 0.012)	0.67 (0.51 - 0.84)*

AUROC – area under the Receiver Operating Characteristic curve; 95%CI - 95% confidence interval.

* p < 0.05 (AUROC = 0.50).

## DISCUSSION

Our study in critically ill elderly patients demonstrates a high prevalence of low muscle mass at the T12 level on CT. Nevertheless, although the muscle mass area on CT scans is associated with BIA variables, these parameters have, at best, a modest accuracy for assessing the skeletal muscle area in critically ill elderly patients. Therefore, our results do not support BIA as a stand-alone tool to exclude low muscle mass in older patients admitted to the ICU.

Some differences between previous studies^(4,8)^ and our report are noteworthy. We studied CT scans at the T12 level, while the previous reports evaluated the L3 level. It is not expected that this difference will bias our results, since there is an excellent correlation between muscle mass areas at T12 and L3 on CT scans.^(9)^ And we only included patients aged ≥ 65 years, while these previous studies enrolled a younger population.

Our study has limitations. First, it was a single-center study, which might limit generalizability. Second, as we previously discussed, we evaluated the T12 level, while most reports used the L3 level. Finally, we enrolled only a minority of admitted patients during the study period, which could introduce selection bias.

The clinical application of our findings is that it would not be possible to exclude the presence of sarcopenia in critically ill elderly patients only with BIA. In these cases, alternative methods for assessing muscle mass are suggested.

## Data Availability

The data that support the findings of this study are available from the corresponding author upon reasonable request.
