# Supplementary material for: Bioelectrical impedance compared to computed tomography for muscle mass evaluation in critically ill elderly patients
Source: Crit Care Sci. 2026 Jul 2;38:e20260004. doi: 10.62675/2965-2774.20260004 (PMC13399239; doi:10.62675/2965-2774.20260004)
Supplement: Supplementary Material [file 2965-2774-ccsci-38-e20260004-suppl1.pdf]

Critical Care Science
https://doi.org/10.62675/2965-2774.20260004

SUPPLEMENTARY MATERIAL

Bioelectrical impedance compared to computed tomography for muscle mass evaluation in critically ill elderly patients

Sandra Regina Alves Belo[1], Naiara Lima Matos[1], Elisa Colonnezi[1], Leandro Utino Taniguchi[1]

INCLUSION AND EXCLUSION CRITERIA

The inclusion criteria were: patients aged ≥ 65 years with a computed tomography (CT) scan made for clinical reasons during intensive care unit (ICU) admission. The exclusion criteria were: inability to perform bioelectrical impedance analysis (BIA) measurement (e.g., severe agitation, presence of prosthetic metal devices); CT images were not suitable for muscle analysis (such as the presence of artifacts, or low resolution or scattering); and BIA acquisition not performed within seven days of CT images.

DATA COLLECTION

During the study period (December 2021 to June 2022), all consecutively admitted patients without exclusion criteria were evaluated. All relevant demographic and outcomes information was retrieved retrospectively from our prospectively collected administrative database (Epimed)[1] and included age, gender, Simplified Acute Physiology Score 3 (SAPS 3),[2,3] type of admission, comorbidities using the Charlson Comorbidity Score,[4] invasive organ support in the ICU, and hospital mortality.

COMPUTED TOMOGRAPHY SCAN ANALYSIS

Computed tomography scans were analyzed by a trained investigator unaware of BIA results using CoreSlicer® (https://coreslicer.com), a validated web-based tool for body composition assessment.[5,6] Computed tomography images were evaluated at the level of the 12th thoracic vertebra (T12), which has exhibited a high correlation with CT at the L3 level[7,8] and has cut-off values for sarcopenia reported for the general population.[9] At the T12 slice, all muscles of the trunk wall, in addition to the erector muscles of the spine, were included.[8] The cross-sectional area (CSA) was measured on a semi-automatic basis after skeletal muscles were visually checked for anatomical features and traced manually. Skeletal muscles were identified using the standard Hounsfield Unit in the range of -29 to +150 Hounsfield units.[5,8] The T12 muscular CSA was automatically calculated by the software and reported in cm^2.

BIOELECTRICAL IMPEDANCE ANALYSIS EVALUATION

Body composition analysis was performed by trained professionals unaware of CT results using a portable segmental BIA device (InBody S10®, Biospace Co. Ltd., South Korea) with 50 kHz alternating current. Bioelectrical impedance analysis-derived measurements included lean body mass (kg), skeletal muscle mass (kg), and phase angle (°). Muscle mass estimation using an equation from Tony Talluri was also used to calculate muscle mass (MMTalluri):[10]

Talluri's equation for total muscle compartment (in kg, where PA is BIA-derived phase angle):

$$\frac{0.3 * fat\ free\ mass * \log(PA)}{0.88} + 0.15 * \left(\frac{total\ body\ water}{0.8} * \log(PA)\right)$$

This is an open access article under the CC BY license (https://creativecommons.org/licenses/by/4.0/).

STATISTICAL ANALYSIS

Continuous variables were tested for normality using the Kolmogorov-Smirnov test. Continuous variables were described as mean (standard deviation) or median (interquartile range [IQR]), when appropriate. Categorical variables were described in terms of frequency and percentages.

Correlation between CT at the T12 level and BIA-derived parameters was estimated using the Spearman correlation method. According to the study by Derstine et al.,[9] we categorized adequate CSA at the T12 level as $\geq 92cm^2$ for males and $\geq 56cm^2$ for females. The discrimination of BIA-derived parameters for identifying patients with adequate muscle mass area at T12 was evaluated using area under the Receiver Operating Characteristic curve (AUROC) analysis. A $p < 0.05$ was considered statistically significant. All statistical tests were conducted two-sided and performed using IBM Statistical Package for the Social Sciences (SPSS), Version 21 (IBM Corp., Armonk, New York, USA) and MedCalc® for Windows, version 19.6 (MedCalc Software, Ostend, Belgium).

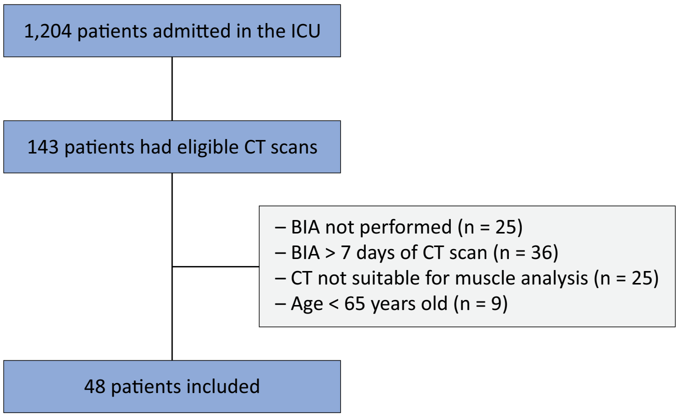

ICU – intensive care unit; CT - computed tomography; BIA - bioelectrical impedance analysis.

Figure 1S - Patients selection process.

Table 1S - General characteristics of participants

	All patients
n	48
Age (years)	81 [74 - 88]
Male	31 (64.6)
SAPS 3	56.5 [46.8 - 62]
SOFA score at ICU admission	4 [2 - 5.75]
BMI (kg/m²)	24.9 [22.7 - 27.9]
Interval between ICU admission and BIA (days)	3 [2 - 5]
Admission type	
Medical	35 (72.9)
Emergency surgery	3 (6.3)
Elective surgery	10 (20.8)
Comorbidities	
Chronic kidney disease	8 (16.7)
Cancer	21 (43.8)
Chronic arterial hypertension	26 (54.2)
Diabetes mellitus	21 (43.8)
Charlson Comorbidity Index	2 [1 - 4]
COVID-19 at ICU admission	9 (18.8)
Mechanical ventilation during ICU stay	19 (39.6)
Vasopressors during ICU stay	25 (52.1)
Renal replacement therapy during ICU stay	5 (10.4)
Hospital mortality	13 (27.1)

SAPS- Simplified Acute Physiology Score; SOFA - Sequential Organ Failure Assessment; ICU - intensive care unit; BMI - body mass index; BIA - bioelectrical impedance analysis. Results expressed as n, median (interquartile range) or n (%).

Table 2S - Computed tomography and bioimpedance information

	All patients
n	48
Weight (kg)	70 [60.5 - 80.0]
CT assessment	
Skeletal muscle area (cm²)	74.5 [61.9 - 90.8]
Low skeletal muscle area*	26 (54.2)
BIA assessment	
Lean body mass (kg)	44.7 [38.1 - 52.0]
Skeletal muscle mass (kg)	25.6 [21.5 - 30.0]
Skeletal muscle mass index (kg/m²)	7.3 [5.7 - 8.5]
Phase angle (degree)	3.2 [2.5 - 3.8]
Muscle mass Talluri equation (kg)	11.5 [9.3 - 15.0]

CT - computed tomography; BIA - bioelectrical impedance analysis. Results expressed as median (interquartile range) or n (%). * Low skeletal muscle area: male < 92cm², female < 56cm².

REFERENCES

1. Zampieri FG, Soares M, Borges LP, Salluh JI, Ranzani OT. The Epimed Monitor ICU Database®: a cloud-based national registry for adult intensive care unit patients in Brazil. Rev Bras Ter Intensiva. 2017;29(4):418-26.
2. Metnitz PG, Moreno RP, Almeida E, Jordan B, Bauer P, Campos RA, et al.; SAPS 3 Investigators. SAPS 3—From evaluation of the patient to evaluation of the intensive care unit. Part 1: Objectives, methods and cohort description. Intensive Care Med. 2005;31(10):1336-44.
3. Moreno RP, Metnitz PG, Almeida E, Jordan B, Bauer P, Campos RA, et al.; SAPS 3 Investigators. SAPS 3—From evaluation of the patient to evaluation of the intensive care unit. Part 2: development of a prognostic model for hospital mortality at ICU admission. Intensive Care Med. 2005;31(10):1345-55.
4. Charlson ME, Pompei P, Ales KL, MacKenzie CR. A new method of classifying prognostic comorbidity in longitudinal studies: development and validation. J Chronic Dis. 1987;40(5):373-83.
5. Mullie L, Afilalo J. CoreSlicer: a web toolkit for analytic morphomics. BMC Med Imaging. 2019;19(1):15.
6. Hey P, Chew M, Wong D, Gow P, Testro A, Kutaiba N, et al. Moving computed tomography-based quantification of muscle mass to the mainstream: validation of a web-based platform to calculate skeletal muscle index in cirrhosis. Liver Transpl. 2022;28(12):1944-6.
7. Nemec U, Heidinger B, Sokas C, Chu L, Eisenberg RL. Diagnosing Sarcopenia on Thoracic Computed Tomography: Quantitative Assessment of Skeletal Muscle Mass in Patients Undergoing Transcatheter Aortic Valve Replacement. Acad Radiol. 2017;24(9):1154 -61.
8. Matsuyama R, Maeda K, Yamanaka Y, Ishida Y, Kato R, Nonogaki T, et al. Assessing skeletal muscle mass based on the cross-sectional area of muscles at the 12th thoracic vertebra level on computed tomography in patients with oral squamous cell carcinoma. Oral Oncol. 2021;113:105126.
9. Derstine BA, Holcombe SA, Ross BE, Wang NC, Su GL, Wang SC. Skeletal muscle cutoff values for sarcopenia diagnosis using T10 to L5 measurements in a healthy US population. Sci Rep. 2018;8(1):11369.
10. Looijaard WG, Stapel SN, Dekker IM, Rusticus H, Remmelzwaal S, Girbes AR, et al. Identifying critically ill patients with low muscle mass: agreement between bioelectrical impedance analysis and computed tomography. Clin Nutr. 2020;39(6):1809-17.
